# Stimulation of the ventromedial prefrontal cortex blocks the return of subcortically mediated fear responses

**DOI:** 10.1038/s41398-022-02174-8

**Published:** 2022-09-20

**Authors:** Christoph Szeska, Hannah Pünjer, Steffen Riemann, Marcus Meinzer, Alfons O. Hamm

**Affiliations:** 1 University of Greifswald, Department of Physiological and Clinical Psychology/Psychotherapy, Franz-Mehring-Strasse 47, 17487 Greifswald, Germany; 2 University Medicine Greifswald, Department of Neurology, Walther Rathenau Str. 49, 17489 Greifswald, Germany

**Keywords:** Long-term memory, Human behaviour, Psychiatric disorders, Physiology

## Abstract

The ventromedial prefrontal cortex (vmPFC) mediates the inhibition of defensive responses upon encounters of cues, that had lost their attribute as a threat signal via previous extinction learning. Here, we investigated whether such fear extinction recall can be facilitated by anodal transcranial direct current stimulation (tDCS). Extinction recall was tested twenty-four hours after previously acquired fear was extinguished. Either anodal tDCS or sham stimulation targeting the vmPFC was applied during this test. After stimulation ceased, we examined return of fear after subjects had been re-exposed to aversive events. Fear was assessed by reports of threat expectancy and modulations of autonomic (skin conductance, heart rate) and protective reflex (startle potentiation) measures, the latter of which are mediated by subcortical defense circuits. While tDCS did not affect initial extinction recall, it abolished the return of startle potentiation and autonomic components of the fear response. Results suggest hierarchical multi-level vmPFC functions in human fear inhibition and indicate, that its stimulation might immunize against relapses into pathological subcortically mediated defensive activation.

## Introduction

The survival of an organism is hinged on its capacity to initiate defensive responses in the face of cues, that have previously been associated with threat—a process, referred to as fear memory recall [1, 2]. Yet, to maintain flexibility, defensive responses likewise decrease, if previous threat cues are no longer associated with harmful events [3, 4]. Such fear extinction is grounded in the establishment of a new extinction memory, that inhibits concomitant fear memory activation upon recall [5, 6]. However, fear extinction recall has been found to be highly context-specific and more fragile compared to well-preserved fear activation [6, 7], entailing a proneness to maladaptive defensive responses, that is particularly pronounced after aversive events have been re-experienced and may eventually result in return of fear [8]. This proneness might even culminate in pathological forms of defensive response activation [9, 10], which is why deficient extinction has been viewed as a risk factor for both pathogenesis and relapses in anxiety, stressor- and trauma-related disorders [11, 12]. Conversely, efficient exposure treatments build upon fear extinction to restore defensive flexibility [5, 13], which is why approaches that may foster the facilitation of extinction recall might be important for promoting the maintenance of therapeutic success [5, 12].

Animal research, that delineated extinction’s neural underpinnings, might provide guidance on how the facilitation of extinction recall and, thus, the prevention of the return of fear can be supported. As was shown, the basolateral complex of the amygdala is a critical site of extinction memory, where the firing of specific extinction neurons invokes an inhibition of the central amygdala that organizes defensive response activation [14, 15]. Whether the established extinction memory is consolidated and eventually recalled during future encounters of the fear cue, however, is dependent on plastic changes in the ventromedial prefrontal cortex (vmPFC) [16], which targets these extinction neurons and additionally inhibits the central amygdala [15, 17–20]. Functional neural imaging research suggested, that the vmPFC exhibits similar inhibitory functions in humans [21–23]. Hence, facilitating vmPFC activity during extinction recall might promote the inhibition of defensive response activation in the amygdala and, thus, prevent return of fear.

Anodal transcranial direct current stimulation (tDCS) is a non-invasive electrical brain stimulation technique that can be used to enhance neural excitability [24–26], thereby allowing to facilitate fear extinction recall in humans using protocols targeting the vmPFC. Surprisingly, however, this hypothesis has never been tested, in spite of the vmPFC’s presumed pivotal role for extinction recall. Previous tDCS research rather focused on increasing vmPFC activity prior to, during, or immediately after extinction learning [27–32]. Accordingly, although these studies demonstrated that preceding or concomitant anodal tDCS may promote initial extinction learning [27, 29, 30, 32], long-term inhibitory effects were rather inconsistent: improved, unaffected, and even impaired extinction recall was found, if anodal tDCS was applied during or after extinction learning [27, 28, 30, 31]. Moreover, these studies primarily measured skin conductance responses as index of fear [27–32]. Skin conductance responses, however, reflect phasic increases in sympathetic arousal as well as orienting and are not specific indices of fear [33], thus, hampering a thorough evaluation of tDCS effects on fear extinction.

**Fig. 1:**
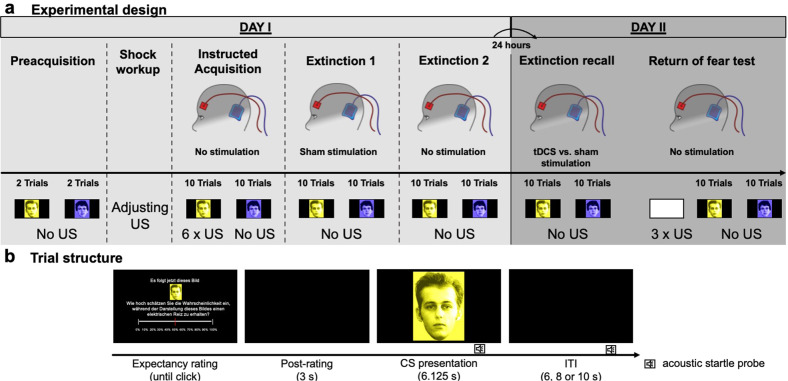
Schematic structure of the experimental design and an experimental trial. **a** Experimental structure. The first day started with a preacquisition phase, where CS+ and CS− were presented twice without any US. Next, the US-intensity was adjusted to be perceived as unpleasant, but not painful by the participant (shock workup). During the following acquisition, first (extinction 1) and second half of extinction training (extinction 2), CS+ and CS− were presented 10 times, each. During acquisition, 6 CS+ trials were paired with the US, while no US was presented during the CS− or during extinction. The second experimental session followed 24 h later and began with an extinction recall phase, where CS+ and CS− were each presented 10 times without any US. A following return of fear test started with a reinstatement procedure, during which the background color of the monitor changed to white and three non-signaled USs were administered. Then, CS+ and CS− were each presented 10 times without the US. TDCS electrodes were always attached aside from preacquisition and shock workup, but electrical currents were only applied during extinction 1 (sham stimulation) and extinction recall (tDCS vs. sham) to minimize contextual effects. **b** Trial structure. A prompting slide required participants to rate the expectancy (in percent range 0–100) to receive a shock during an upcoming CS presentation (﻿English translation: “Next, this picture will follow. How likely do you think is it, to receive an electrical shock during the upcoming presentation of this picture?”). After a three-second post-rating interval, the CS was presented full-size, followed by an inter-trial interval (ITI). Startle probes were administered during CSs and ITIs.

**Fig. 2:**
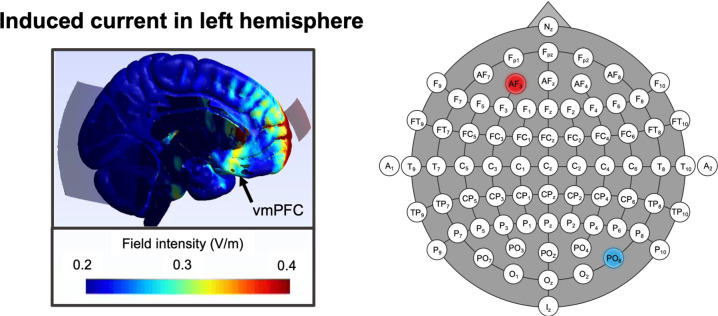
Biophysical modeling of the current’s distribution and magnitude induced by anodal tDCS applying a 3 × 3 cm² anode over AF_3_ and a 10 × 10 cm² cathode over PO_8_. The left panel depicts changes in electrical field intensity (V/m) when tDCS is applied in the described montage, while the right panel depicts the according location of the anode (red) and cathode (blue) according to the 10–20 EEG system (see “Methods” for details on biophysical modeling).

To close the current research gap, we used a more comprehensive multi-level approach of fear assessment and examined the effects of a stimulation protocol that targeted the vmPFC during extinction recall in a sham-controlled and double-blinded between-group design. To this end, forty participants completed a two-day differential cue extinction paradigm, that involved two conditioned stimuli (CS), of which one (CS+) signaled the occurrence of an aversive unconditioned stimulus (US), while another stimulus did not (CS-; for details see also Fig. 1a and “Methods”). Twenty-four hours after a thus established differential fear response was ought to be extinguished, we tested the recall of extinction memory while participants either received a sham stimulation or active anodal tDCS, that invoked a strong and focal activation of the vmPFC as suggested by previous research [29] and our own current modeling [34] (Fig. 2; see “Methods” for details). After the stimulation ceased, a final return of fear test followed, during which we tested the persistence of fear attenuation after aversive events (i.e., the US) have been re-experienced. Throughout the experiment, fear was measured by higher-order cognitive [35–38], as well as low-level autonomic and reflex indices of defensive response activation [39–42]. Here, ratings of the probability, with which the occurrence of US is expected during the upcoming CS, indicated cognitive threat expectancy (see also Fig. 1b). Skin conductance responses indicated sympathetic arousal, which has previously been found to be mediated by amygdala activity [33, 41, 43]. Furthermore, two additional indirect read-outs of amygdala activity were measured: the potentiation of the startle reflex (*fear potentiated startle*) and cardiac deceleration (*fear bradycardia*; see “Methods” for a detailed description) [39–41, 44–47]. These defensive response components reflect fear-induced behavioral freezing in rodents [48, 49] and are related to attentive immobility [44, 45, 50–53]—the main defense strategy during fear conditioning in both humans and non-human animals, ensuring a high degree of translational validity [54].

Given that the vmPFC has been ascribed a pivotal role in the consolidation and retrieval of extinction memory, we hypothesized that an anodal transcranial direct current stimulation targeting the vmPFC during extinction memory retrieval would facilitate extinction recall and prevent a subsequent return of fear. Since the vmPFC has found to exhibit such fear-attenuating effects by top-down inhibition of the amygdala, we further expected that these effects would be particularly pronounced for amygdala-dependent indicators of defensive responding (i.e., skin conductance, fear potentiated startle and fear bradycardia) [33, 41, 43, 55]. On the other hand, we did not expect similar inhibitory effects for cognitive indices of fear activation, which have been suggested to be mediated by various cortical and hippocampal areas [35, 37, 42].

## Methods

### Participants

To determine an appropriate sample size to test our hypotheses with sufficient power (i.e., ≥0.80) [56], we conducted an a-priori power analysis using G*Power [57, 58] that was based on previous research demonstrating large inhibitory effects of vmPFC firing on defensive responding [17, 21]. Our analysis revealed, that a sample of 36 participants is required to detect such large inhibitory effects (i.e., *f*^*2*^ ≥ 0.35) [56] with sufficient power, if linear regression approaches ought to be used as in the current study (for more information see “Statistical analysis and figure creation”). In addition, the sample size estimation was also guided by (a) previous studies that demonstrated significant beneficial stimulation effects on different aspects of fear extinction (*N* = 12–44) [27–31] and, (b) our own studies that demonstrated beneficial effects of tDCS in between subjects (single and multisession) designs (*N* = 28–50) [59–62].

Forty-two students of the University of Greifswald were recruited. Two participants were excluded from the analyses due to premature termination of the experiment, resulting in a final sample of 40 participants (mean age = 21.85, range = 18–27 years; 38 women; 3 left-handed). Inclusion criteria embraced an age between 18–35 years and a body-mass-index in the normal range (18.5–27 kg/m^2^). Previous or current medical or mental conditions, that would have affected any of the outcome measures or would have conflicted with electrical stimulation, led to an exclusion. This comprised any previous or current neurological condition (e.g., stroke, epilepsy, migraine, multiple sclerosis, tumors), cardiovascular condition (e.g., hypertension, heart attacks, wearing artificial heart valves), other bodily condition (e.g., diabetes, neurodermatitis on the hand’s palm, hormonal disorders, impaired vision or hearing), the wearing of implants (e.g., pacemakers, cochlea implants), pregnancy, but also any previous or current mental disorder, as well as the previous or current use of psychotropic drugs. The applicability of in- and exclusion criteria was assessed on the basis of participants’ self-report during a phone interview. Each participant gave her/his informed consent and either received partial course credits or monetary reward (25 €). The study was approved by the ethical committee of the University Medicine Greifswald.

### Experimental design

In our study, we applied anodal tDCS in a sham-controlled between-group design, embracing differential-cue fear acquisition and extinction procedures on two separate experimental days (for a schematic overview see Fig. 1a). Prior to the experimental manipulation, eligible participants were randomly allocated to either an active anodal (*n* = 20) or a sham tDCS (*n* = 20), determining whether anodal tDCS or sham stimulation was delivered during extinction recall (day II). At this, group allocation was double-blinded (for details see “Transcranial direct current stimulation”). Throughout the experimental stages, two different conditioned stimuli (CS; colored pictures) and startle probes were presented [4.5 s or 5 s after CS/inter-trial interval (ITI) onset; mean probe onset for both trial types = 4.65 s]. The order of stimulus presentation was arranged in two experimental versions, that subjects were randomly assigned to and that were counterbalanced across participants. Throughout the entire experiment, subjects sat in a sound-attenuated and dimly-lit chamber, adjacent to the experimenters room.

### Day I

After a startle habituation period, during which six acoustic startle probes were presented (inter-stimulus intervals of 9, 11, 12, 6, and 10 s; *M* = 9.6 s) to adapt participants’ startle magnitudes to a stable baseline, the first experimental day started with a preacquisition phase. Here, the CS+ and CS− were each presented twice. No aversive unconditioned stimuli (US) were applied, which the experimenter explicitly communicated to the subjects beforehand, assisted by explicitly showing that the electrode for US-delivery had not been attached yet. During all CS-trials and two inter-trial intervals (ITI), startle probes were administered to assess a baseline of startle reflex magnitudes before any learning task or any aversive stimulation has taken place.

After preacquisition, the electrode for US-delivery was attached to the participants non-dominant hand’s wrist and subjects underwent a shock workup procedure, during which the US intensity was adjusted to a level, that was perceived as unpleasant, but not painful. To this end, a number of sample shocks were presented, beginning at an amperage of 2 mA. After each administration of the electric stimulus, participants rated the perceived shock intensity on a continuous 5-point visual analog scale, that ranged from “1 (not painful)” to “5 (very painful)”. Depending on the rating by the participant, the experimenter increased or decreased the shock intensity for the upcoming trial following a standardized protocol [53, 63]. The shock workup continued, until a shock intensity was rated as “4 (unpleasant)”, which was then set for the experiment.

Next, we attached the electrodes for the application of tDCS, however, without any stimulation being delivered. In that, we aimed at making the experimental context as comparable as possible for all learning tasks, thus, minimizing contextual effects on fear acquisition, extinction and extinction recall. Furthermore, we thus made sure that participants would not elaborate tDCS electrodes as a safety signal, as would have resulted if they were only attached during extinction tasks. After fitting the tDCS-electrodes, participants underwent an instructed fear acquisition training. Here, subjects were instructed, that USs may be delivered, but only during the presentation of the CS+. Importantly, however, no information was given with regard to the explicit contingency between the CS+ and the US. During this phase, the CS+ and CS− were presented 10 times, with only the former being paired with the US in six trials (60% CS-US contingency). Startle probes were administered in eight of the CS+ and eight of the CS− trials as well as in eight ITIs.

Next, a sham stimulation was started for all subjects, determined to last 20 min and, thus, including the following first half of the extinction training (extinction 1). In that, we aimed at providing a similar tingling sensation during extinction training and recall (day II) and, thus, aimed at abolishing noticeable contextual differences between extinction learning and retrieval. During the first half of the extinction training (extinction 1), participants were only informed that previously presented stimuli *might* be administered again. Here, both the CS+ and CS− were each presented 10 times, while no USs were delivered. Startle probes were, again, administered in eight of the CS+ and eight of the CS− trials as well as in eight ITIs.

After the first half of the extinction training, a short experimental break was implemented, that allowed the stimulation to finish and, thus, made sure that no stimulation was delivered during the upcoming second half of the extinction training (extinction 2). Here, without further instructions, stimulus presentation was kept similar to the first half of the extinction training.

### Day II

The second experimental day started 24 h after day I. First, tDCS stimulation electrodes were applied and the stimulation was started, determined to last 20 min, thus, including the following extinction recall phase, during which participants were only informed that any of the previously presented stimuli might be presented again. At this, depending on the participant’s stimulation condition, subjects either received anodal tDCS or a sham stimulation.

During the extinction recall, which started off with a startle habituation phase similar to the first day, the CS+ and CS− were each presented 10 times, while no USs were presented. Startle probes were administered in eight of the CS+ and eight of the CS− trials as well as in eight ITIs.

After the extinction recall phase, a short experimental break was implemented to allow the stimulation to finish, thus, ensuring that no stimulation was delivered during the upcoming return of fear test. Without further instructions being given, the return of fear test started off with a reinstatement procedure, during which three non-signaled USs were delivered without any concomitant CS presentation. Afterwards, the CS+ and CS− were each presented 10 times, while no USs were presented. Startle probes were, again, administered in eight of the CS+ and eight of the CS− trials as well as in eight ITIs.

### Stimulus materials

Selected stimulus materials are depicted in Fig. 1b. Two colored pictures of male faces with neutral facial expressions from the Psychological Image Collection at Stirling [64, 65] were used as CS+ and CS− (counterbalanced across participants). Visual CSs were presented for a duration of 6.25 s on a black background via a 24-inch monitor (1024 × 768 pixel resolution), that was set 1.45 m in front of the participant. During ITIs, only the black background was presented (6, 8 or 10 s; mean duration = 8 s), whose color changed to white during the reinstatement procedure (30 s) to prevent counter-conditioning to the ITI.

An electric shock was used as an unconditioned stimulus (US), that was administered by an S-48K stimulator (Grass instruments, West Warwick, RI, USA) to the subject’s non-dominant hand’s wrist. This electric shock comprised a train (500 ms) of 100 single electrical pulses, each with a duration of 2 and 3 ms inter-stimulus interval. The intensity of the US was individually adjusted for each subject to be perceived as unpleasant, but not painful during the shock workup. No differences in US-intensity was observed between stimulation conditions (*M*_tDCS_ = 2.98, SD = 0.74; *M*_Sham_ = 2.65, SD = 0.99; Stimulation, *F*_(1,38)_ = 1.381, *P* = 0.247).

A 95 dB(A) burst of white noise with an instant rise/fall time (<1 ms), that was binaurally presented via AKG K66 headphones for 50 ms, served as acoustic startle probe to elicit the startle eyeblink.

### Transcranial direct current stimulation

Anodal transcranial direct current stimulation was delivered using a one-channel, battery-driven direct current stimulator (DC-Stimulator Plus, NeuroConn, Ilmenau, Germany). Two electrodes were placed in reusable sponge pockets, that were saturated with 0.9% saline and were attached by rubber headbands to the subjects’ skulls. The anode was placed over the left frontal cortex (position AF_3_ according to 10–20 EEG system) while the cathode was placed over the parieto-occipital cortex (position PO_8_ according to 10–20 EEG system), thus, closely following previous research examining the impact of tDCS on fear extinction [29].

Electrode size was determined by simulations using SimNIBS software [34]. Since no individual structural MRIs were available, a head mesh based on the MNI brain was used to calculate electric fields with the finite element method. Electric field simulations were computed based on different combinations of common anode and cathode sizes (3 × 3, 3 × 5, 5 × 5 cm² and 5 × 5, 10 × 10 cm² respectively) at AF_3_ and PO_8_. During all simulations electrodes were oriented horizontally. The sponge and electrode thickness were set at 5 and 2 mm, respectively. Tissue conductivities remained at SimNIBS default values. The electrical fields were visually inspected to pick the montage with the highest current intensities in the vmPFC while keeping the current to a minimum in surrounding areas. Thus determined final montage embraced a 3 × 3 cm^2^ anode and a 10 × 10 cm^2^ cathode (Fig. 2). Using this montage, active stimulation was administered with 2 mA, which was applied for 20 min. Sham stimulation was administered only for 30 s with 2 mA. In both stimulation conditions, stimulation ramped up to 2 mA for 8 s and ramped down for 5 s. Stimulation conditions did not differ in average contact quality/impedance (Stimulation, *F*_(1,38)_ = 1.845, *P* = 0.182).

Based on previous research, we expected that active and sham tDCS would result in a comparable tingling, yet transient scalp sensation, that fades out after a brief period of time either because the stimulation is ramped down (sham tDCS) or because the participant habituates to the stimulation (active tDCS) [66]. In fact, this is a well-established setup to ensure participant blinding [66–68]. To verify the blindness of the experimental subjects to the allocated stimulation, we assessed their awareness to stimulation by questionnaires, that required the participant to report, at which experimental day active tDCS had been delivered. The majority of participants reported, that active anodal stimulation had been delivered during both experimental days. Only one subject correctly reported, that active stimulation was applied only during day II.

To additionally ensure blindness of the experimenter to the applied stimulation protocol, we used the so-called “study mode” of the tDCS device. This mode requires the experimenter to enter an individual code to start the stimulator, which was determined prior to the experiment and encoded the designated stimulation protocol (i.e., active or sham tDCS). Importantly, the respective code for each participant was assigned by a researcher not involved in the experimental procedures to ensure blinding of the experimenter administering the stimulation.

### Assessments and data reduction

#### Startle eyeblink response

The eyeblink component of the startle reflex, that was elicited by the acoustic startle probe, was measured by two electrolyte-filled (Marquette Hellige, Freiburg, Germany) Ag/AgCl miniature surface electrodes (3 mm diameter, Sensormedic, Yorba Linda, CA, USA), recording the electromyographic activity of the orbicularis oculi muscle underneath the left eye. A Coulbourn S75–01 system amplified and filtered the EMG signal with a 30 Hz high-pass and a Kemo LEM-VBF8-03 400 Hz low-pass filter, that smoothed the rectified signal with a time constant of 10 ms. An additional 50 Hz notch filter was used to eliminate 50-Hz interference. The EMG data were digitally sampled at a rate of 1000 Hz and a computer program [69] detected startle eyeblink responses semi-automatically between 100 ms before and 400 ms after the startle probe onset. The detected startle response was then visually inspected for artifacts and pre-determined onset and peak were manually corrected if necessary. Startle responses were finally scored, if they started between 20–120 ms and peaked within 150 ms after the startle probe administration with a minimum amplitude of 1.954 μV. While trials were scored as zero responses if no blink was detected (mean during day 1: 3.5%, mean during day 2: 4.3%), trials were scored as missing (mean during day 1: 6.4%, mean during day 2: 9.35%), if clear movement artifacts or excessive baseline activity hampered the evaluation of responses [70]. After the scoring procedure, raw startle blink magnitudes were standardized by a z-transformation and a subsequent T-transformation (50 + (z × 10)), which was performed individually for each participant and separately for each experimental day in order to adjust for individual differences in overall startle magnitude. Finally, we computed startle potentiation scores by subtracting T-standardized CS startle magnitudes from T-standardized ITI startle magnitudes on a trial-by-trial basis. Standardized potentiation scores for each trial were then averaged in blocks of two trials to compensate for missing values.

### Skin conductance response

The skin conductance was measured at the participant’s non-dominant hand by two electrolyte filled (0.05 M sodium chloride) Ag/AgCl electrodes (8 mm diameter) that were attached on the hypothenar eminence of the palmar surface. A Coulbourn S71–22 skin conductance coupler, providing a constant current of 0.5 V across the two electrodes, amplified the signal, which was digitally sampled at a rate of 10 Hz and processed with a resolution of 0.001 µS. The first interval skin conductance response (FIR) was finally determined by scoring the maximal change in skin conductance (minimum amplitude of 0.01 µS), which started between 0.9–4 s after stimulus onset. Trials in which no response could be detected were scored as zero responses (mean during day 1: 63.8%, mean during day 2: 69.5%), while trials with recording artifacts were scored as missing (mean during day 1: 1.3%, mean during day 2: 0.4%). After scoring, logarithms of the skin conductance responses (adding the constant 1 to every value) were computed to normalize skin conductance data, that is regularly skewed in extinction studies due to the high rate of zero responses [71]. To further reduce interindividual variability that was not related to the learning tasks, log scores were range corrected by dividing each individual score by the participant’s maximum response within all CS and US trials, separately for each day [71, 72]. For final analysis, range-corrected values were, just like startle responses, averaged across blocks of two trials.

### Heart rate

Cardiac responding was measured by two electrolyte-filled (Marquette Hellige, Freiburg, Germany) Ag/AgCl electrodes (8 mm diameter), that were applied in an Einthoven Lead II setup for electrocardiography. A Coulbourn V75-04 system filtered the signal with an 8–13 Hz band-pass filter and amplified the signal by the factor 2000, which was then digitally sampled at 400 Hz and corrected for artifacts using ANSLAB (v. 2.4; Autonomic Nervous System Laboratory, University of Basel, Switzerland). After artifact correction, the ECG data was converted to heart rate in beats per minute for every half-second of the sampling period [73]. Allowing a quantification of baseline-independent conditioned cardiac responding, heart rate during CS presentations was subtracted from a base period heart rate (mean of the first two half-seconds before CS onset) for every half-second of the CS duration (13 data points for the 6.125 s duration of the CS). Finally, the resulting half-second based difference scores (Δ-bpm) were averaged across all trials of an experimental stage (preacquisition, acquisition, extinction 1, extinction 2, extinction recall, return of fear test).

### Shock expectancy ratings

Before each CS presentation, participants were informed that a CS+ or CS− will be displayed in the upcoming trial, assisted by a presentation of the respective CS in smaller size (Fig. 1b). Before CSs were presented, however, participants were required to rate their expectancy of receiving an electrical shock during the upcoming CS. To this end, they shifted a red cursor on a continuous 11-point visual analogue scale (ranging from “0%” to “100%”) and pushed the left mouse button to log in the rating. Only after the rating was logged, a three-second post-rating period (black screen) followed. Then, the CS was presented in full size. On the one hand, the design of this rating procedure ensures that no cognitive evaluation task interferes with physiological responses during the CS trial. More importantly from a clinical perspective, however, this rating procedure closely adapts to exposure therapy, during which patients are asked to evaluate the probability, that their central concern (e.g., a dog bite) might become true before the exposure takes place [65, 74, 75]. Finally, rating data was averaged across blocks of two trials.

### Statistical analysis and figure creation

Linear mixed effect models were used to analyze the between-group fixed-effect of Stimulation (tDCS vs. sham stimulation) on defensive responding towards either conditioned Stimulus (CS+ vs. CS−; within-subject fixed-effect), separately for each experimental phase (see Fig. 1a). Additionally taking into account the temporal dynamics of conditioned responding across blocks of two averaged trials, these models further included the repeated-measures fixed-effect *Block* for startle, skin conductance and US-expectancy rating data (1–4 blocks for startle potentiation, 1–5 blocks for SCR and US-expectancy rating data during each phase). Taking into account the temporal dynamics of averaged cardiac responding during the CS presentation, the repeated-measures fixed-effect Time was entered into analysis (1 to 13 half-second bins during CS presentation). For all analyses, level of statistical significance was set at *p* < 0.05.

Linear mixed models were chosen, as they may increase the statistical power of analysis for two reasons: First, missing values in the subject’s data do not lead to an exclusion of the case and second, error covariance structures in repeated measure designs may be modeled according to theoretical assumptions [76, 77]. To this end, all models applied a restricted maximum likelihood estimation (REML) to include all available data [77]. Moreover, as the factors *Block* and *Time* imply dependence of repeated measure observations, a first-order autoregressive (AR1) covariance structure was modeled, which assumes that adjacent observations are statistically more closely related compared to more distant ones [78]. Statistical analyses were performed by IBM SPSS 28. Figures were created by Microsoft Excel and PowerPoint as well as Adobe Illustrator.

## Results

### Establishment and extinction of the fear response

**Fig. 3:**
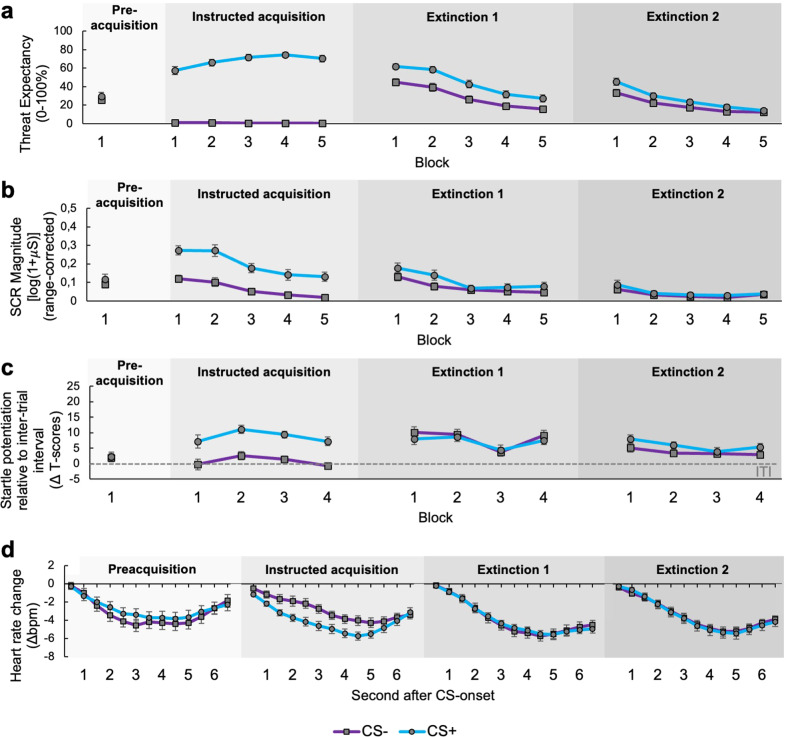
Day 1: Establishment and extinction of the fear response. Overall mean US-expectancy ratings (**a**), range-corrected first interval skin conductance responses (**b**), standardized (T-transformed) startle potentiation (**c**; response levels above 0 show a relative potentiation of the startle responses relative to the ITI control condition; note the relative increase in startle magnitudes probed during CS− from instructed acquisition to non-instructed extinction) and heart rate change (**d**) during the CS+ (blue lines) vs. the CS− (purple lines) during preacquisition, instructed fear acquisition as well as the first (extinction 1) and second half of extinction training (extinction 2). US-expectancy, skin conductance and startle potentiation are averaged across blocks of two trials, while heart rate change is averaged across all trials of a respective phase. Error bars represent the standard error of the mean.

While during preacquisition defensive response activation did not differ between both designated CSs (all *F*s ≤ 3.120, all *P*s ≥ 0.085; Fig. 3), threat expectancy (Fig. 3a), skin conductance responses (Fig. 3b), startle potentiation (relative to the inter-trial interval, ITI; Fig. 3c) and cardiac deceleration (Fig. 3d) significantly increased in the face of the CS + relative to the CS- during the instructed acquisition training (treat expectancy, skin conductance and startle potentiation: Stimulus, all *F*s ≥ 52.548, all *P*s < 0.001; Fig. 3a–c; heart rate: Stimulus × Time, *F*_(12,882.020)_ = 2.122, *p* = 0.014; Fig. 3d). This indicates a well-established differential fear response on multiple response levels (also see Supplemental Material for details). As expected, we did not find any significant differences between the designated tDCS and sham stimulation groups during the instructed acquisition training in any fear measure (all *F*s ≤ 3.758, all *P*s ≥ 0.054; Fig. 3), indicating that the robust differential fear response was acquired independent of the upcoming stimulation conditions (see Supplemental Material for details).

During the subsequent extended extinction training (extinction 1 and 2), defensive responses during the CS− somewhat increased, suggesting increased defensive sensitization to the safety-signaling stimulus, possibly as a result of increased uncertainty due to missing instructions regarding the US-occurrence (see Supplemental Material for details). Nevertheless, fear responses to both CSs continuously decreased (see Supplemental Material for details). As a result, threat expectancy and skin conductance did no longer differ between CS+ and CS− (Stimulus, all *F*s ≤ 1.074, all *P*s ≥ 0.307; Fig. 3a, b) and dropped below preacquisition levels (Block, all *F*s ≥ 11.774, all *P*s < 0.001) during the final block of extinction 2, indicating successful extinction on these response levels. In contrast, startle reflexes were still potentiated during CSs relative to the ITI (Intercept, *F*_(1,38.251)_ = 16.449, *P* < 0.001; Fig. 3c) during the final extinction block, and such potentiation was significantly higher when blinks were evoked during the CS+ relative to the CS− (Stimulus, *F*_(1,37.601)_ = 6.310, *P* = 0.016; Fig. 3c). Moreover, fear bradycardia was also still evident (Time, *F*_(12,852.020)_ = 29.313, *P* < 0.001), but did no longer discriminate between CS+ and CS− during extinction 2 (Stimulus × Time, *F*_(12,900.506)_ = 0.250, *P* = 0.995; Fig. 3d). Hence, the data suggest that subcortically mediated indices of fear, that are related to attentive immobility, were not fully extinguished and also showed some sustained generalization towards the CS−. Importantly, however, stimulation groups did not differ in their fear responses during the second half of extinction (all *F*s ≤ 1.414, all *P*s ≥ 0.154; Fig. 3), suggesting a comparable partial fear extinction prior to the second experimental day (see Supplemental Material for details).

### Transcranial direct current stimulation over the vmPFC does not impact on initial extinction recall

**Fig. 4:**
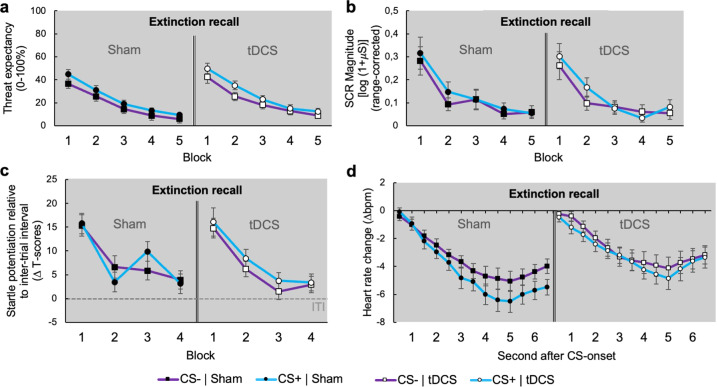
Day 2: Transcranial direct current stimulation targeting the vmPFC does not impact on initial extinction recall. Overall mean US-expectancy ratings (**a**), range-corrected first interval skin conductance responses (**b**), standardized (T-transformed) startle potentiation relative to inter-trial interval (**c**) and heart rate change (**d**) during the CS+ (blue lines) vs. the CS− (purple lines) during the extinction recall for the sham (left panels) and tDCS condition (right panels). US-expectancy, skin conductance and startle potentiation are averaged across blocks of two trials, while heart rate change is averaged across all trials of the extinction recall. Error bars represent the standard error of the mean.

The second experimental session started twenty-four hours later with a test of extinction memory recall, during which half of the participants received anodal tDCS targeting the vmPFC, while the other half received sham stimulation. In both stimulation conditions, all fear indices started at an elevated level compared to the last block of extinction on the previous day (Block, all *F*s ≥ 73.204, all *P*s < 0.001; Block × Stimulation, all *F*s ≤ 2.764, all *P*s ≥ 0.100; Figs. 3a, b, c and 4a, b, c). This effect was equally pronounced for both conditioned stimuli (Stimulus x Block, all *F*s ≤ 1.764, all *P*s ≥ 0.187), indicating a strong overall defensive sensitization. While US-expectancy ratings settled on an elevated level during the CS+ relative to the CS− (Stimulus, *F*_(1,197.265)_ = 5.513, *P* = 0.020; Fig. 4a), skin conductance, startle potentiation and still-evident cardiac deceleration (Time, *F*_(12,842.926)_ = 26.311, *P* < 0.001; Fig. 4d) did not significantly differ between both cues throughout extinction recall (all *F*s ≤ 3.145, all *P*s ≥ 0.077; Fig. 4b–d), suggesting a well-established CS + -US memory on a cognitive level, but a more generalized defensive activation in amygdala driven fear indices.

Despite the successive reduction of the overall response magnitudes during extinction recall (Block, all *F*s ≥ 26.496, all *P*s < 0.001; Fig. 4a–c), threat expectancy during the CS + remained elevated compared to the CS− even during the last block of extinction recall (Stimulus, *F*_(1,38)_ = 4.793, *P* = 0.035; Fig. 4a), while skin conductance and startle potentiation remained on a comparable level for both conditioned stimuli (Stimulus, all *F*s ≤ 0.223, all *P*s ≥ 0.639; Fig. 4b, c). During the final block of extinction recall, however, latter two fear response components finally reached a level that was comparable to preacquisition, indicating partly successful recall of extinction memory for these amygdala driven indices of fear (skin conductance: Block, *F*_(1,148.888)_ = 3.809, *P* = 0.053; startle potentiation: Block, *F*_(1,141.552)_ = 0.611, *P* = 0.436; Fig. 4b, c). Unexpectedly, tDCS had no effect on extinction recall in any of the measured outcomes (all *F*s ≤ 2.602, all *P*s ≥ 0.053; Fig. 4).

### Transcranial direct current stimulation targeting the vmPFC blocks the return of subcortically mediated fear responses

**Fig. 5:**
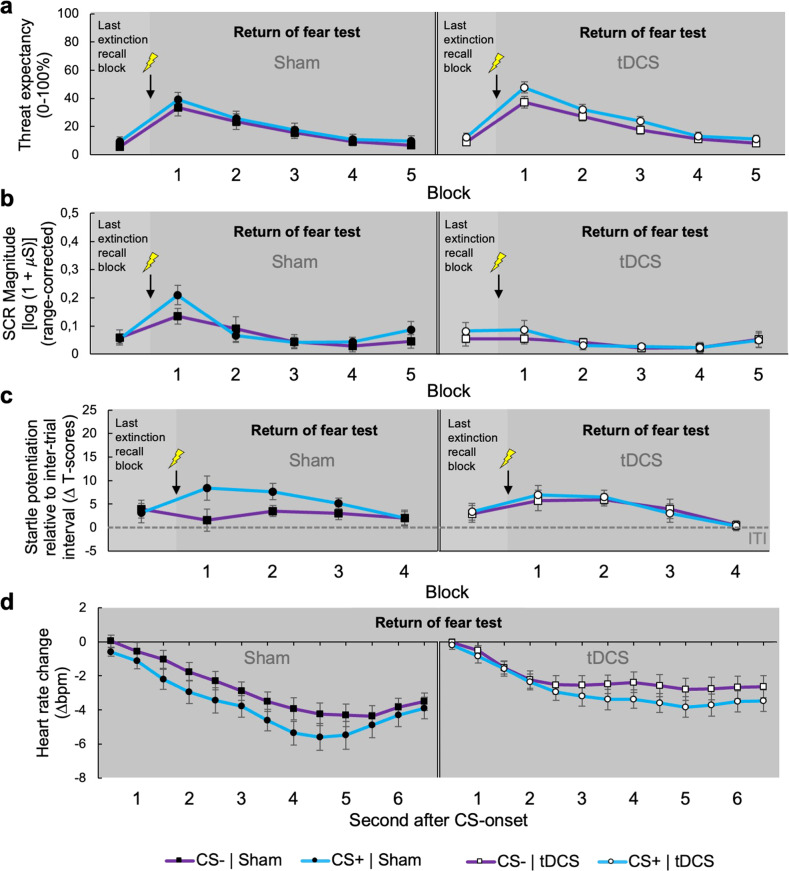
Day 2: Transcranial direct current stimulation targeting the vmPFC during extinction recall blocks the return subcortically mediated fear responses. Overall mean US-expectancy ratings (**a**), range-corrected first interval skin conductance responses (**b**), standardized (T-transformed) startle potentiation relative to inter-trial interval (**c**) and heart rate change (**d**) during the CS+ (blue lines) vs. the CS− (purple lines) during the last block of extinction recall and return of fear test for the sham (left panels) and tDCS condition (right panels). US-expectancy, skin conductance and startle potentiation are averaged across blocks of two trials, while heart rate change is averaged across all trials of the return of fear test. Error bars represent the standard error of the mean. The bolt-icon represents repeated (three times) administration of the US at the beginning of the return of fear test.

Importantly, however, the application of tDCS during the extinction recall significantly impacted on the following return of fear test, during which stimulation was no longer applied. This final experimental phase started with the administration of three non-signaled USs, typically leading to a reinstatement of fear [8]. This was indeed the case for threat expectancy, which similarly increased for both CSs from the last extinction recall block to the first block of the return of fear test (Block, *F*_(1,91.029)_ = 198.697, *P* < 0.001; Stimulus × Block, *F*_(1,116.062)_ = 0.843, *P* = 0.361; Fig. 5a). At this, we could not observe any differences between both stimulation conditions (Block × Stimulation, *F*_(1,91.029)_ = 0.599, *P* = 0.441; Stimulus × Block × Stimulation, *F*_(1,116.062)_ = 0.283, *P* = 0.596; Fig. 5a), suggesting that previous tDCS did not affect fear reinstatement on a cognitive level. Supporting this notion, we did not observe any differences in threat expectancy between both stimulation conditions throughout the entire return of fear test (all *F*s ≤ 0.831, all *P*s ≥ 0.367; Fig. 5a). In contrast, previous tDCS substantially attenuated the reinstatement of fear as indexed by response components that are mediated by the amygdala.

In the sham condition, skin conductance responses significantly increased from the last extinction recall block to the first block of the return of fear test during both CSs (Block, *F*_(1,36.542)_ = 26.252, *P* < 0.001; left panel of Fig. 5b). Thus, the CS+ again evoked stronger skin conductance responses compared to the CS− immediately after the reinstatement procedure (Stimulus, *F*_(1,19)_ = 5.399, *P* = 0.031; left panel of Fig. 5b). Likewise, fear potentiated startle was reinstated to the CS+ in sham subjects immediately after the re-experience of the US (Stimulus, *F*_(1,18)_ = 5.026, *P* = 0.038; left panel of Fig. 5c). Such reinstated fear potentiated startle responses during the CS + even maintained throughout the entire return of fear test in sham subjects (Stimulus, *F*_(1,71.833)_ = 5.855, *P* = 0.018; left panel of Fig. 5c). In contrast, after active tDCS, repeated presentations of the US did not lead to an increase in skin conductance responses to either CS (Block, *F*_(1,32.139)_ = 0.009, *P* = 0.925; right panel of Fig. 5b). In fact, skin conductance responses were attenuated in tDCS relative to the sham subjects throughout the entire return of fear test (Stimulation, *F*_(1,105.064)_ = 8.856, *P* = 0.004; Fig. 5b). Likewise, the tDCS condition did not show immediate (Block, *F*_(1,33.615)_ = 3.615, *P* = 0.066; Stimulus × Block, *F*_(1,54.673)_ = 0.049, *P* = 0.825; right panel of Fig. 5c) or maintained reinstatement of fear potentiated startle responses during the CS+ (Stimulus, *F*_(1,63.117)_ = 0.039, *P* = 0.845; right panel of Fig. 5c). Moreover, cardiac deceleration to both CSs was significantly less pronounced in subjects, that received active tDCS during the foregoing extinction recall, compared to the sham condition (Stimulation × Time, *F*_(12,841.430)_ = 2.004, *P* = .021; Fig. 5d). Thus, tDCS during extinction recall has blocked the return of low-level fear activation after the repeated experience of the threat cue.

## Discussion

The activation of the ventromedial prefrontal cortex (vmPFC) has been ascribed a pivotal role in the mediation of fear extinction recall, as it drives the inhibition of amygdala-dependent defensive responses in the face of extinguished threat signals [15–19]. In this study, we examined whether anodal transcranial direct current stimulation (tDCS) targeting the vmPFC facilitates such fear extinction recall in humans, as indexed by cognitive threat expectancy [38], but also by autonomic (skin conductance response, fear bradycardia) and behavioral (fear potentiated startle) fear indices, that serve as indirect read-outs of amygdala activity [33, 39, 40, 43–47]. In contrast to our hypothesis, tDCS did not impact on defensive responses during initial extinction recall. However, after tDCS ceased and subjects were re-exposed to the original threat stimuli, the effects of foregoing tDCS unfolded: In subjects that had received sham tDCS, such re-exposure evoked a generalized defensive re-activation to both the previous threat and safety signal and also led to sustained return of fear towards the previous fear cue. Active tDCS that targeted the vmPFC abolished both generalized defensive re-activation but also cue-specific fear reinstatement for low-level, subcortically mediated components of the defensive response pattern. Only cognitive threat expectancy remained unaffected.

While the results therefore appear somewhat conflicting with the concept, that vmPFC activity is necessary for extinction recall [19, 79], they might indeed call for a refinement of this view. Specifically, animal research suggests that successful retrieval of extinction memory is in fact hinged on two vmPFC-dependent processes—first, the consolidation and second, the recall of extinction memory. Extinction consolidation has found to be mediated by NMDAR-dependent plastic changes in the vmPFC, which make a subsequent inhibition of the amygdala (i.e., extinction recall) possible in the first place [15, 16]. It is therefore tempting to speculate, that a manipulation of vmPFC activity preferably impacts on pending extinction consolidation before recall can be affected at all, even though both processes might run in concert to allow parallel updating and retrieval of extinction memory. This view is supported by animal findings, which showed that silencing of the vmPFC during extinction learning impairs long-term fear inhibition, while silencing during extinction recall does not impact on retrieval processes, possibly because in both cases a pending (re-)consolidation of extinction memory has been disrupted [80]. Our results extend this picture of hierarchical multi-level vmPFC functioning to human fear inhibition and indicate, that tDCS targeting the vmPFC has first and foremost facilitated a pending re-consolidation of extinction memory after previous partial fear extinction learning, which— perhaps in combination with offset-effects that facilitated subsequent recall—immunized against the return of fear.

As this effect was particularly pronounced for low-level amygdala-dependent defensive responses, it was likely driven by inhibition of the amygdala via increased vmPFC signaling. Hence, our data suggest comparable functions of the vmPFC in animal and human fear extinction [15, 19]. Inhibited amygdala signaling, however, might be less important for an inhibition of fear-related cognition, which depends on an assembly of neural structures involving the hippocampus and various cortical areas rather than the amygdala [35, 42]. Supporting this view, cognitive threat expectancy has not been affected by a stimulation of the vmPFC in our study. Thus, although our data indicate that tDCS of the vmPFC might stabilize extinction of maladaptive low-level defense, the vmPFC might be less of a promising target if cognitive indices of fear activation ought to be attenuated.

Hence, our results might help to re-evaluate the vmPFC as a stimulation target for the facilitation of exposure treatment. Such treatment builds upon extinction learning and serves as an efficient therapeutic regimen for anxiety, stressor- and trauma-related mental disorders [5, 13, 74, 81]. However, mitigated vmPFC activity in these patients [11] might drive deficient extinction [82] and thus contribute to treatment non-responding and particularly relapses of fear [83]. Hence, it has been suggested that a stimulation of the vmPFC might cope with extinction deficits and promote exposure effects [29, 84]. Refining this view, our results indicate that a stimulation of the vmPFC after exposure might not influence immediate extinction memory recall (i.e., retrieval of the exposure experience), but might rather impact on the long-term inhibition of low-level defensive responses and thereby contribute to the prevention of relapses into pathological defensive response activation. Importantly, as suggested by our data, this protective effect against low-level defensive re-activation seems not restricted to a single fear cue but might also generalize to similar cues that were not previously associated with the threat. Indeed, preliminary evidence from patients with PTSD suggests, that stimulation of the vmPFC during exposure does not reduce cognitive fear ratings [84], but attenuates PTSD symptoms that are related to defensive response outputs (e.g., startle potentiation, sweating) in the face of trauma-related cues [29]. The current data suggest, that stimulation of the vmPFC during an additional rehearsal session after successful exposure might even better protect against relapses.

In sum, the current results therefore not only refine our understanding of the vmPFC’s role in human fear inhibition, but also define future areas of application, where vmPFC stimulation might help to improve exposure therapy.

### Limitations

Nevertheless, it has to be acknowledged that our study used a highly controlled experimental approach to investigate the fear-attenuating effects of anodal tDCS targeting the vmPFC during extinction recall. This involved, that only healthy participants have been examined, possibly limiting the generalizability of results to clinical populations. Moreover, this also involved that experimental contexts were kept similar between fear acquisition, extinction learning, extinction recall and the return of fear test in order to validly assess fear responses that are independent of contextual effects. In clinical practice, however, it is often not possible to conduct exposure treatments in the same context, where fear has originally been established, e.g., war zones in case of veterans with PTSD. Likewise, return of fear may occur in a context that significantly differs from the setting that was embraced in exposure therapy. As such contextual changes may significantly draw on the long-term attenuation of fear [85, 86], future research should test, whether the current inhibitory effects of anodal tDCS targeting the vmPFC not only hold in clinical populations, but also under varying environmental circumstances.

## Supplementary information

Supplemental Material

## Data Availability

Data and code, that were used in this study, is available upon request from the corresponding author.
