# Supplementary material for: Stimulation of the ventromedial prefrontal cortex blocks the return of subcortically mediated fear responses
Source: Transl Psychiatry. 2022 Sep 20;12:394. doi: 10.1038/s41398-022-02174-8 (PMC9489865; doi:10.1038/s41398-022-02174-8)
Supplement: Supplementary file 1 — Supplemental Material [file 41398_2022_2174_MOESM1_ESM.docx]

**Supplemental Material**

During preacquisition, we found non-zero threat expectancy (Intercept, F_(1,38)_ = 47.595, *P* < .001; **Fig. 3a**) and skin conductance (Intercept, *F*_(1,38)_ = 26.891, *P* < .001; **Fig. 3b**), as well as a significant cardiac deceleration during both designated CSs (Time, *F*_(12,829.728)_ = 7.960, *P* < .001; **Fig. 3d**). Startle responses during CSs were not significantly potentiated relative to the inter-trial interval (ITI; Intercept, *F*_(1,36.914)_ = 3.608, *P* = .065; **Fig. 3c**). As expected, none of these responses significantly differed between the two stimuli (all *F*s ≤ 3.120, all *P*s ≥ .085; **Fig. 3**). Thus, results indicate innate but equally expressed cognitive and physiological defensive activation to both cues.

In comparison to preacquisition, we found substantially stronger increases in threat expectancy (Stimulus x Block, *F*_(1,127.067)_ = 50.663, *P* < .001; **Fig. 3a**), skin conductance (Stimulus x Block, *F*_(1,121.053)_ = 8.290, *P* = .005; **Fig. 3b**) and startle potentiation (Stimulus x Block, *F*_(1,127.932)_ = 4.533, *P* = .035; **Fig. 3c**) to the CS+ relative to the CS- during the first block of acquisition training. As a result, the CS+ evoked stronger defensive responses compared to the CS- throughout the entire fear acquisition training (Stimulus, all *F*s ≥ 52.548, all *P*s < .001; **Fig. 3a, b, c**), indicating a well-established differential fear response on multiple levels of expression. Accordingly, the CS+ also evoked a stronger cardiac deceleration compared to the CS- (Stimulus x Time, *F*_(12,882.020)_ = 2.122, *p* = .014; **Fig. 3d**). As expected, we did not find any significant differences between the designated tDCS and sham stimulation groups during the instructed acquisition training in any of the outcomes (all *F*s ≤ 3.758, all *P*s ≥ .054; **Fig. 3**), indicating that differential fear responses were equally acquired across both stimulation conditions.

With the beginning of the subsequent non-instructed extinction training (first block of extinction 1), threat expectancy, skin conductance and startle potentiation during the CS- increased relative to the last acquisition block, suggesting an increased defensive sensitization to the safety-signaling stimulus, possibly as a result of increased uncertainty due to missing instructions regarding the US-occurrence (Block, all *F*s ≥ 24.516, all *P*s ≤ .001; **Fig. 3a, b, c**). Despite such defensive sensitization, however, the CS+ continued to evoke higher threat expectancy compared to the CS- throughout extinction 1 (Stimulus, *F*_(1,234.023)_ = 23.541, *P* < .001; **Fig. 3a**), indicating that threat expectancies were well-elaborated. Consolidated defensive activation was also observed on both the physiological and behavioral levels, as skin conductance and startle potentiation during the CS+ was comparable between the first block of extinction 1 and the end of acquisition (skin conductance: Block, *F*_(1,38)_ = 2.809, *P* = .102; startle potentiation: Block, *F*_(1,37.945)_ = .134, *P* = .716; ; **Fig. 3b, c**). However, due to elevated responses during the CS-, neither skin conductance (Stimulus, *F*_(1,138.983)_ = 3.854, *P* = .052; **Fig. 3b**) nor startle potentiation significantly differed between both CSs during extinction 1 (Stimulus, *F*_(1,141.113)_ = .668, *P* = .415; **Fig. 3c**), suggesting that defensive sensitization towards the safety-signaling cue was particularly pronounced for low-level indices of defensive responding. Accordingly, strong cardiac deceleration (Time, *F*_(12,849.725)_ = 37.913, *P* < .001) was equally expressed during both CSs during extinction 1 (Stimulus x Time, *F*_(12,891.882)_ = .371, *P* = .974; ; **Fig. 3d**).

Nevertheless, shock expectancy, skin conductance as well as startle potentiation started to decrease during extinction 1 (Block, all *F*s ≥ 5.026, all *P*s ≤ .002; **Fig. 3a, b, c**). Such decrease continued during the second half of the extinction training (extinction 2) for threat expectancy and autonomic arousal (Block, all *F*s ≥ 4.583, all *P*s ≤ .001; **Fig. 3a, b**), while startle potentiation remained on a stable level (Block, *F*_(3.218.923)_ = 2.562, *P* = .056; **Fig. 3c**). Thus, the data reflect a reduction of defensive response activation throughout extinction training, although behavioral fear expression was stronger preserved. As a result of reduced defensive response activation, threat expectancy and skin conductance during the final block of extinction 2 no longer differed between CS+ and CS- (Stimulus, all *F*s ≤ 1.074 , all *P*s ≥ .307; **Fig. 3a, b**) and were even lower compared to preacquisition (Block, all *F*s ≥ 11.774, all *P*s < .001), indicating successful extinction learning on these response levels. In contrast, we found higher startle potentiation during the CS+ relative to the CS- during the final extinction block (Stimulus, *F*_(1,37.601)_ = 6.310, *P* = .016; **Fig. 3c**). Moreover, fear bradycardia was still evident (Time, *F*_(12,852.020)_ = 29.313, *P* < .001) and equally pronounced during both conditioned stimuli throughout the entire extinction 2 (Stimulus x Time, *F*_(12,900.506)_ =.250, *P* = .995; **Fig. 3d**). Hence, the data suggest that fear towards the CS+ as well as defensive sensitization during the CS-, as expressed by behavioral and physiological indices of attentive immobility, was not fully extinguished. Nonetheless, there was no difference in defensive responding between both stimulation groups during the second half of extinction (all *F*s ≤ 1.414, all *P*s ≥ .154; **Fig. 3**), suggesting a comparable partial fear extinction prior to the second experimental day.
